# Supplementary material for: MetaQTL: a package of new computational methods for the meta-analysis of QTL mapping experiments
Source: BMC Bioinformatics. 2007 Feb 8;8:49. doi: 10.1186/1471-2105-8-49 (PMC1808479; doi:10.1186/1471-2105-8-49)
Supplement: Additional File 7 — MetaQTL Package : jar file and tutorial. This Zip archive contains both the MetaQTL JAR file and the files of the tutorial. [file 1471-2105-8-49-S7.zip › org.inra.metaqtl/doc/index.html]

Generated Documentation (Untitled)


<H2>
Frame Alert</H2>
<P>
This document is designed to be viewed using the frames feature. If you see this message, you are using a non-frame-capable web client.
<BR>
Link to<A HREF="overview-summary.html">Non-frame version.</A>
